# Supplementary material for: Lignin-Modified Single-Use Graphite Electrodes: Electrochemical Detection of DNA, Mitomycin C, and Their Interaction
Source: Sensors (Basel). 2025 Sep 2;25(17):5427. doi: 10.3390/s25175427 (PMC12431613; doi:10.3390/s25175427)
Supplement: Supplementary file 1 [file sensors-25-05427-s001.zip › sensors-3801114-supplementary.pdf]

Supplementary Material

Instruments

A µAUTOLAB electroanalysis system (Eco Chemie, 2014, Utrecht, The Netherlands) controlled by NOVA 1.11 software was used for DPV and CV analyses. EIS and SFI analyses were performed using an AUTOLAB 204 PGSTAT with FRA 2.0 module controlled by NOVA 2.1 software (Eco Chemie, The Netherlands). FE-SEM and EDX analysis were performed with ZEISS Gemini SEM 500.

All measurements were performed in a three-electrode system in a Faraday cage. In the three-electrode system, an Ag/AgCl/3.0 M KCl electrode (BAS, Model RE-5B, W. Lafayette, USA) was used as the reference electrode, platinum wire as the auxiliary electrode, and a disposable pencil graphite electrode (PGE) as the working electrode. The pencil was held vertically with 14 mm of the lead protruding outside, 10 mm of which was immersed in the solution.

Chemicals

Lignin (LG), Mitomycin (MC), fish sperm double stranded DNA (fsDNA), N-(3-Dimethylaminopropyl)-N′-ethylcarbodiimide hydrochloride (EDC), N-Hydroxysuccinimide (NHS), and other chemical agents were purchased from Sigma-Aldrich. All chemicals were of analytical reagent grade, and Milli-Q ultra-pure water was used in all solutions.

The stock solution of fsDNA was prepared at a concentration of 1000 µg/mL using ultra-pure water, and it was kept at -20 °C. fsDNA was diluted with acetate buffer solution (ABS, 0.5 M, pH 4.8). The stock solution of MC was prepared at a concentration of 1250 µg/mL using ultra-pure water, and it was kept at +4 °C. MC was diluted with phosphate buffer solution (PBS, 0.05 M, pH 7.4). The covalent agent solution (CA) was prepared fresh daily with PBS (0.05 M, pH 7.4) containing 5 mM EDC (1-Ethyl-3-(3-dimethylaminopropyl)carbodiimide)/8 mM NHS (N-Hydroxysuccinimide) and kept at +4 °C.

Electrochemical Measurements

Voltammetric measurements

CV measurements were carried out −0.5 to + 1.0 V at the scan rate of 50 mV/s in 5 mM $[Fe(CN)_6]^{3-/4-}$ (1:1) prepared with PBS (0.05 M, pH 7.4) containing 0.1 M KCl as a redox probe solution.

DPV measurements were carried out 0 to + 0.4 V potential at 50 mV pulse amplitude and 50 mV/s scan rate in 5 mM $[Fe(CN)_6]^{3-/4-}$ (1:1) prepared with PBS (0.05 M, pH 7.4) containing 0.02 M NaCl as a redox probe solution.

Impedimetric measurements

EIS measurements were carried out under the frequency 0.1 to 100000 Hz (50 logarithmic equal intervals) and a voltage of 0.01 V amplitude at an open circuit potential of +0.23 V in 1 mM $[Fe(CN)_6]^{3-/4-}$ (1:1) containing 0.1 M KCl as a redox probe solution.

An equivalent circuit model, the Randles circuit, was used to fit the impedance data. The Randles circuit includes C, constant phase; W, Warburg impedance element; R_s, solution resistance; and R_{ct}, charge transfer resistance. The relevant semicircle diameter corresponds to R_{ct}. The corresponding circuit model fits the data obtained from the EIS measurements; this is shown in the Nyquist diagrams. All Nyquist diagrams were fitted by the relevant data.

A

Element	Weight %	Atomic %	Net Int.	Error %	Kratio	Z	A	F
C K	90.89	94.46	7988.49	5.01	0.5647	1.0075	0.6167	1.0000
O K	4.43	3.45	163.05	14.84	0.0047	0.9622	0.1106	1.0000
AlK	0.43	0.20	151.29	6.79	0.0031	0.8536	0.8454	1.0078
SiK	4.18	1.86	1631.46	2.41	0.0341	0.8720	0.9315	1.0047
S K	0.07	0.03	23.07	27.74	0.0006	0.8535	0.9814	1.0114

B

Element	Weight %	Atomic %	Net Int.	Error %	Kratio	Z	A	F
C K	89.74	93.55	9532.78	4.99	0.5564	1.0081	0.6150	1.0000
O K	5.45	4.27	246.77	13.76	0.0059	0.9628	0.1121	1.0000
NaK	0.35	0.19	75.04	12.11	0.0016	0.8729	0.5163	1.0024
AlK	0.40	0.19	169.60	6.71	0.0029	0.8542	0.8380	1.0075
SiK	3.97	1.77	1863.03	2.42	0.0322	0.8726	0.9261	1.0047
S K	0.09	0.04	34.19	25.64	0.0008	0.8540	0.9808	1.0113

Figure S1. EDX spectrum: (A) PGE (B) PGE/LG.

Table S1. Average current values (*n* = 3) obtained by CV of Lignin modified PGEs prepared with different solvents, and control groups.

**Solvent**	**Electrodes**	**I_a (μA)**	**RSD (%)**	**% Change in current value**
		**0.28 V**		
**DMF**	***PGE***	263.05 ± 5.77	2.19	**-**
	PGE/LG	271.08 ± 16.51	6.09	**3% increase**
**DMSO**	***PGE***	242.23 ± 4.61	1.90	**-**
	PGE/LG	283.27 ± 3.69	1.30	**17% increase**

**% change values were calculated based on the average current values (n = 3) obtained with control groups (PGE).*

Table S2. Average current values (*n* = 3) obtained by CV of LG modified electrodes at different concentrations.

LG concentrations (μg/mL)	I_a (μA) 0.28 V	RSD (%)	% Change in current value*
PGE	242.23 ± 4.61	1.90	-
**250**	248.95 ± 13.32	5.35	**3% increase**
**500**	283.27 ± 3.69	1.30	**17% increase**
**750**	291.24 ± 2.03	0.70	**20% increase**
**1000**	279.68 ± 4.38	1.57	**15% increase**
**1500**	276.76 ± 10.68	3.86	**14% increase**
**3000**	263.10 ± 4.88	1.85	**9% increase**

**% change values were calculated based on the average current value (n = 3) obtained with control group (PGE).*

Table S3. Average current values (*n* = 3) obtained by CV of LG modified electrodes at different times.

LG modification time (min)	Electrodes	I_a (μA) 0.28 V	RSD (%)	% Change in current value*
**30**	***PGE***	242.23 ± 4.61	1.90	-
	PGE/LG	291.24 ± 2.03	0.70	**20% increase**
**60**	***PGE***	258.21 ± 7.83	3.03	-
	PGE/LG	276.04 ± 6.77	2.45	**7% increase**

**% change values were calculated based on the average current values (n = 3) obtained with control groups (PGE).*

Table S4. Average current values (*n* = 3) obtained by CV after EDC/NHS activation of LG modified electrodes for different times.

CA activation time (min)	Electrodes	I_a (μA) 0.28 V	RSD (%)	% Change in current value*
**5**	***PGE***	243.25 ± 4.40	1.81	-

	PGE/LG	264.37 ± 3.00	1.13	**9% increase**
**30**	***PGE***	236.90 ± 16.18	6.83	**-**
	PGE/LG	263.23 ± 7.46	2.83	**11% increase**
**60**	***PGE***	242.23 ± 4.61	1.90	**-**
	PGE/LG	291.24 ± 2.03	0.70	**20% increase**

**% change values were calculated based on the average current values (n = 3) obtained with control groups (PGE).*

Table S5. Optimum conditions determined for electrode preparation.

Variable	Optimum Condition
LG solvent	DMSO
Lg solution concentration	750 µg/mL
LG modificaiton time	30 minutes
CA activation time	60 minutes

Table S6. The average values (*n* = 3) of the anodic peak current (I_a), anodic charge value (Q_a), and the calculated surface area (A) obtained by PGE control and PGE/LG.

Electrodes	I_a (µA)	Q_a (mC)	A (cm^2)
PGE control	242.23 ± 4.61	2.54 ± 0.09	**0.292**
PGE/LG	291.24 ± 2.03	2.83 ± 0.06	**0.352**

Table S7. Average R_{ct} values (*n* = 2) obtained and calculated apparent fractional coverage values by EIS of PGE control and PGE/LG before and after 0.01 µg/mL fsDNA immobilization.

Electrodes	Average R_{ct} (ohm)	RSD (%)	% Change in R_{ct} value	Apparent fractional coverage value
PGE	901.50 ± 78.49	8.71	-	-
PGE/fsDNA	983.50 ± 37.48	3.81	9% increase	**0.083**
PGE/LG	489.00 ± 15.56	3.18	-	**-**
PGE/LG/fsDNA	1415.00 ± 91.92	6.50	189% increase	**0.654**

**% change values were calculated based on the average R_{ct} values (n = 2) obtained with control groups (PGE and PGE/LG).*

Table S8. Average current values (*n* = 3) obtained by DPV of PGE control and PGE/LG before and after 0.01 µg/mL fsDNA immobilization.

Electrodes	I_a (µA)	RSD (%)	% Change in current value*
	0.2 V		
PGE	215.83 ± 4.89	2.27	**-**
PGE/fsDNA	207.40 ± 7.95	3.83	**4% decrease**
PGE/LG	308.92 ± 3.32	1.07	**-**
PGE/LG/fsDNA	279.06 ± 5.03	1.80	**10% decrease**

**% change values were calculated based on the average current values (n = 3) obtained with control groups (PGE and PGE/LG).*

Table S9. Average current values (*n* = 3) obtained by DPV after immobilization of PGE/LG with 20 µg/mL fsDNA for different times.

Electrodes	fsDNA immobilization time	I_a (µA)	RSD (%)	%Change in current value
		0.20 V		
PGE/LG	**-**	308.92 ± 3.32	1.07	**-**
PGE/LG/fsDNA	***15 min.***	194.13 ± 5.34	2.75	**31% decrease**
	30 min.	151.57 ± 10.09	6.66	**46% decrease**
	60 min.	162.37 ± 5.51	3.39	**43% decrease**

**% change values were calculated based on the average current values (n = 3) obtained with control group (PGE/LG) optimum experimental conditions.*

Table S10. Average current values (*n* = 3) and %RSD values obtained by DPV after immobilization of different concentrations of fsDNA on PGE/LG.

fsDNA concentrations (ng/mL)	I_a (µA)	RSD (%)
	0.2 V	
**Blank (PGE/LG)**	308.92 ± 3.32	1.07
**10^1**	279.06 ± 5.03	1.80
**10^2**	234.01 ± 11.08	4.73
**10^3**	201.11 ± 10.46	5.20

**10^4**	158.23 ± 10.99	6.95
**10^5**	116.13 ± 9.46	8.15
**10^6**	128.37 ± 3.54	2.76

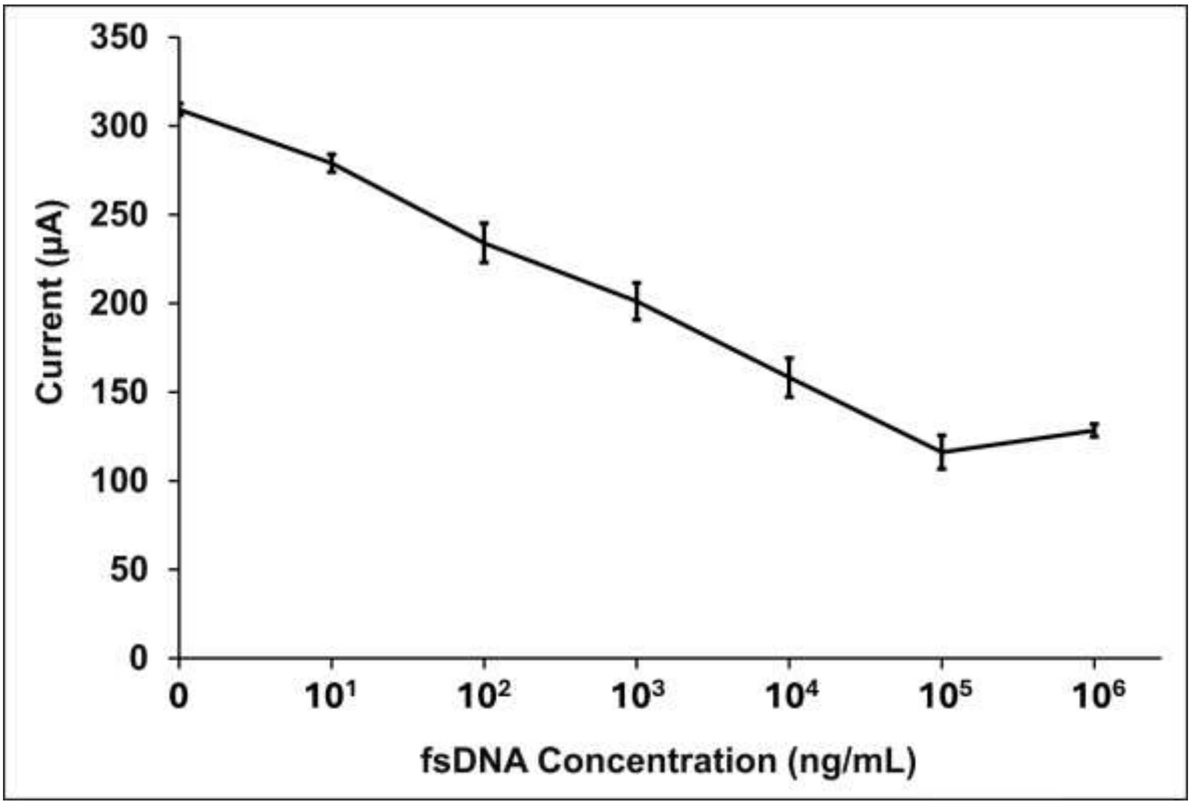

Figure S2. Line graph showing the average current values measured after immobilization of fsDNA concentrations in the range 0- 10^6 ng/mL using PGE/LG electrodes (*n* = 3).

Table S11. Average current values (*n* = 3) and %RSD values obtained by DPV after immobilization of different concentrations of MC on PGE/LG.

MC concentratioons (pg/mL)	I_a (µA)	RSD (%)
	0.2 V	
**Blank (PGE/LG)**	308.92 ± 3.32	1.07
**10^0**	287.97 ± 3.19	1.11
**10^1**	255.09 ± 3.97	1.56
**10^2**	231.03 ± 19.50	8.44
**10^3**	205.15 ± 10.23	4.98
**10^4**	177.38 ± 7.02	3.96
**10^5**	163.37 ± 4.14	2.53
**10^6**	135.02 ± 13.57	10.05
**10^7**	224.34 ± 13.81	6.16

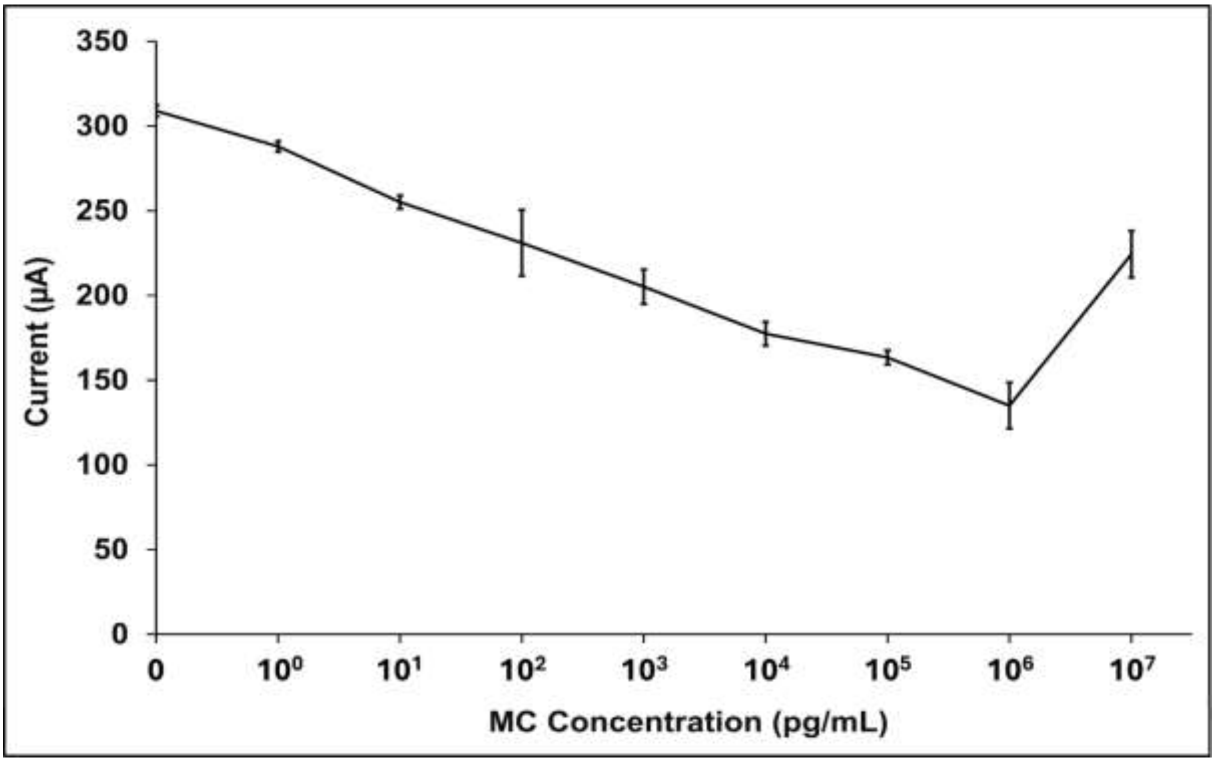

Figure S3. Line graph showing the average current values measured after immobilization of MC concentrations in the range 0-10^7 pg/mL using PGE/LG electrodes (*n* = 3).

Limit of detection

In this study, the limit of detection was calculated according to the IUPAC method [1]. The analyte's signal at the detection limit (Sdl) is given by:

Sdl = Sreag + k * σreag,

where Sreag is the electrochemical signal for a blank, σreag is the known standard deviation for the blank's electrochemical signal (n_σ=3)

k is a numerical factor chosen according to the confidence level desired. In this study k value was selected as k=3 (99.86 % confidence level) according to the Long and Winefordner [2].

The electrochemical signal response values (I_a) used to calculate LOD were presented in Table S9 and Table S10 for fsDNA and MC, respectively. The developed biosensor's detection limit was calculated as 2.95 ng/mL for fsDNA and 0.22 pg/mL for MC by the data given in Table S9 and Table S10, formula and calibration curves.

Table S12. Average redox probe signals (*n* = 3) and decrease rates of MC and DNA obtained by DPV before and after interaction at different times.

Interaction time	Redox probe signal of MC (µA)	Redox probe signal of fsDNA (µA)

(min)	Before interaction	After interaction	Decrease %	Before interaction	After interaction	Decrease %
15	274.00 ± 24.10 %RSD: 8.79 %	115.09 ± 1.89 %RSD: 1.64 %	**58**		115.09 ± 1.89 %RSD: 1.64 %	**24**
30	238.88 ± 24.25 %RSD: 10.15 %	101.71 ± 1.81 %RSD: 1.78 %	**57**	151.57 ± 10.09 RSD: 6.66 %	101.71 ± 1.81 %RSD: 1.78 %	**33**
60	234.03 ± 14.83 %RSD: 6.34 %	123.13 ± 16.00 %RSD: 12.99%	**47**		123.13 ± 16.00 %RSD: 12.99%	**19**

Table S13. Average R_{ct} values (*n* = 2) of MC and DNA obtained by EIS before and after interaction at different times.

Interaction time (min)	Redox probe signal of MC before interaction (ohm)	Redox probe signal of fsDNA before interaction (ohm)	Redox probe signal after interaction (ohm)
15	716.50 ± 17.68 %RSD: 2.47 %		1995.00 ± 134.35 %RSD: 6.73 %
30	958.00 ± 12.73 %RSD: 1.33 %	1625.00 ± 35.36 %RSD: 2.18 %	2615.00 ± 63.64 %RSD: 2.43 %
60	881.50 ± 19.09 %RSD: 2.17 %		1700.00 ± 113.14 %RSD: 6.66 %

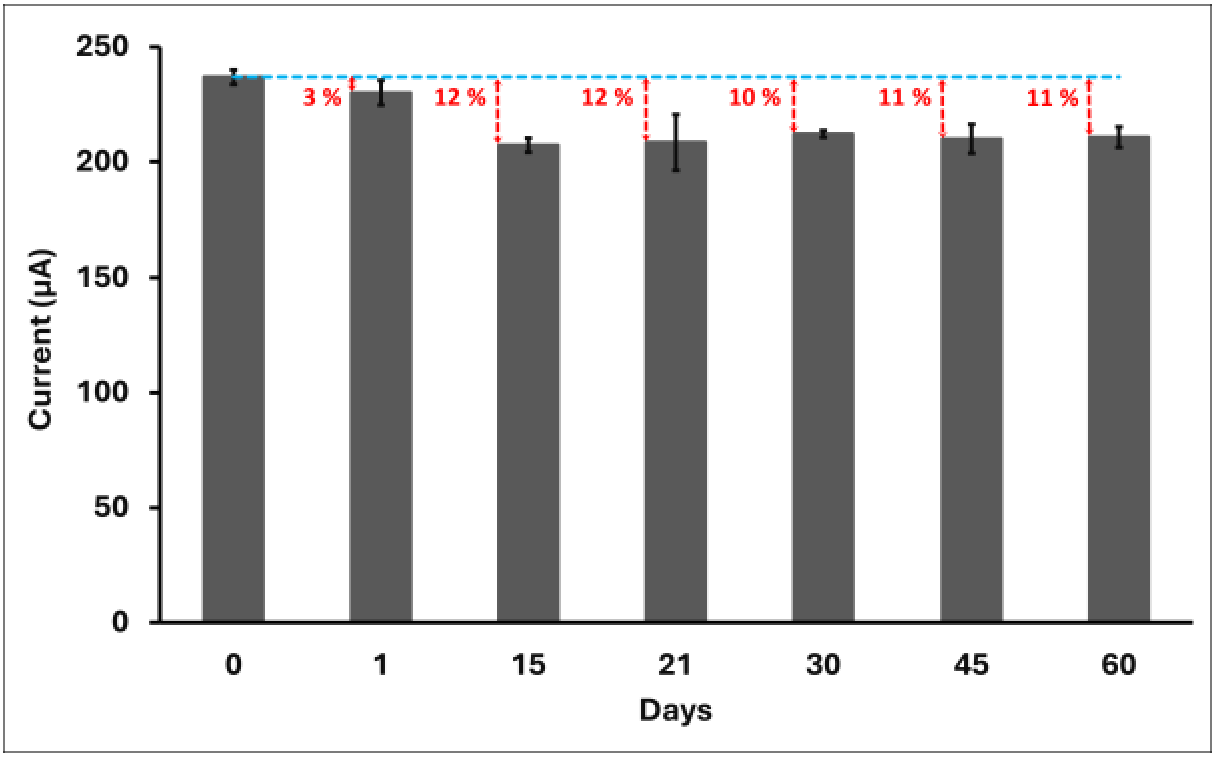

Figure S4. Stability of the developed biosensor in storage (*n = 3*).

References

1. IUPAC Nomenclature, Symbols, Units and Their Usage in Spectrochemical Analysis—III. Analytical Flame Spectroscopy and Associated Non-Flame Procedures. *Pure Appl. Chem.* **1976**, *45*, 105–123.
2. G.L. Long, J.D. Winefordner, Limit of Detection A Closer Look at the IUPAC Definition, Anal. Chem. ***1983***, 712–714.
